# What’s Economics Got to Do with It? Providing Theoretical Clarity on ELSA of AI

**DOI:** 10.1007/s11948-025-00564-x

**Published:** 2025-11-14

**Authors:** Mark Ryan, Vincent Blok

**Affiliations:** 1 Wageningen Social and Economic Research, Pr. Beatrixlaan 582 - 528, Den Haag, 2595 BM The Netherlands; 2 Philosophy Group, Wageningen University & Research, P.O. Box 8130, Wageningen, 6700 EW The Netherlands

**Keywords:** AI ethics, Artificial intelligence, Economics, Ethical, legal, and social aspects, Industry

## Abstract

While research in the ethics of artificial intelligence (AI) has grown recently, the relationship between AI’s ethical and economic dimensions is under-researched. This is surprising, given the considerable investments in AI by Big Tech companies (e.g., Microsoft, META and IBM) and their ambiguous role in today’s public debate on AI. After the second Trump election, this ambiguity has resulted in industry opposition to rules and regulations (e.g., disinvestments in moderation facilities at social media platforms and calls for deregulation). AI ethics must respond to the economic underpinnings of this situation.

While economics in AI ethics has also been seen in recent funding schemes (e.g., investment in 30 ethical, legal, and social (ELSA) labs), there is a ambiguity in how these AI ELSA labs should respond to economic aspects. This paper examines the role of economics in responsible AI research, using the case of the ELSA lab approach. The four features of ELSA (proximity, anticipation, interdisciplinarity, and interactivity) serve as a point of departure to demonstrate how economics can be integrated within the ELSA framework of AI. This paper proposes that economics should be integrated within these four ELSA features to implement responsible AI successfully.

## Introduction

While research in the ethics of AI has grown significantly in the past few years, the relationship between AI’s ethical and economic aspects is relatively under-researched to date. This lack of research is surprising, given the considerable investments in Research & Development on AI in the private sector (e.g., Big Tech like Microsoft, META and IBM) and the ambiguous role private sector actors play in today’s public debate on AI. For example, in the open letter to pause research (Future of Life Institute, 2023), governments were called to install rules and regulations to limit irresponsible AI. At the same time, many Big Tech companies (e.g., OpenAI) have heavily lobbied against the European AI Act (Perrigo, 2023). Following the second Trump election in the United States (US), this ambiguity has even led to opposition from private sector actors against ethical rules and regulations (Fitzpatrick & Ottenberg, 2025). We see, for instance, various forms of disinvestment in moderation facilities on social media platforms, the privatisation of public data in the US, and calls for more deregulation, including ethical regulation, for enterprises (Gross, 2024; Özdemir, 2024). There is a tension between ethical and social requirements and economic aspects (Blok and Lemmens 2015). Therefore, it is becoming clear that the ethics of AI research need to understand and respond to these economic aspects in the real world for it to be effective.

The importance of considering economics within the ethics of AI research has also been seen in many recent funding schemes; for example, the investment in 30 ELSA ‘labs’ focusing on artificial intelligence (AI) (funding through the AINed of the Netherlands AI Coalition (Van Veenstra, van Zoonen, & Helberger, 2021). The ELSA labs are large projects (€2.2 million, five years, with approximately 20 researchers involved in each project) focusing on the ELSA of different domains and applications of AI technologies (e.g., defence, agri-food, poverty, media, and health) (Brightlands Smart Services Campus, 2024) (Nederlandse AI Coalitie 2024). These labs aim to ensure AI’s ‘societal readiness levels’ and ‘pathways to impact’ and incorporate public values (Van Veenstra, van Zoonen, & Helberger, 2021). They require the inclusion of stakeholders to ensure that ‘the socio*economic* impact of AI becomes manageable and people will be confident about how it works’ (Nederlandse AI Coalitie 2024) [emphasis ours].

Despite this, a structural tension remains between ethics and economics in developing responsible AI in practice, and there is an ambiguity about how these ELSA labs should respond to economic aspects. Even if we strongly argue for the primacy of responsible AI over economics in AI research (like the ELSA labs), we still have to take the economics in which ethical, legal and social aspects of AI emerge, and should be addressed, more seriously. Otherwise, this leaves these ELSA labs and ELSA researchers (in the Netherlands labs alone, there will be approximately 600 researchers by 2027)^1^ with the challenge of how to include economics in their projects.

In this paper, we aim to open a debate on the tensions between the ethics and economics of AI by exploring the role of economics in responsible AI research, using the case of the ELSA lab approach. This paper begins by consulting the literature on ELSA to identify the role of economics in current ELSA research. It will become clear that little attention is given to economics within current ELSA literature. While some authors argue that economics should be included in ELSA research (Ryan & Blok 2023; Zwart et al., 2014), the literature lacks guidance on where economics fits within ELSA research. It is unclear how economics relates to ‘ethical’, ‘legal’, or ‘social’ aspects or whether it needs to be at all.

For this reason, we continue in Sect. “What Should Be the Role of Economics in ELSA of AI?” with a critical reflection on the role of economics in ELSA research. The four features of ELSA (proximity, anticipation, interdisciplinarity, and interactivity) (Zwart & Nelis 2009) are taken as a point of departure to demonstrate how economics can be integrated within an ELSA of AI approach. Section four focuses on whether economics should be included as a fourth pillar of ELSA of AI research. While it proposes that some arguments against its inclusion are insufficient, we conclude that economics should not be included as a fourth pillar because it would distort the original intention of ELSA so much as to make the approach meaningless and contradictory to its raison d’être. Overall, economics can and should be integrated within the four ELSA features to achieve better alignment between responsible AI and economics, while maintaining the core features and objectives of AI research.

## What Is ELSA, and What Does it Say About Economics?

To answer the question ‘What is the role of economics in ELSA?’, it is essential to understand what ELSA means and what current ELSA research has to say about it. ELSA research originated from a need to better embed social science analysis into science and technology. It was a fundamental component of the 4th European Research Framework Programme (in 1994). It was a European response to address challenges and issues in science and technologies, with a specific emphasis on the life sciences (e.g., human genomics) and stems from its US counterpart – ELSI research (instead of ‘aspects’, it refers to ‘impacts’ (Fisher, 2005; Lewenstein & Brossard 2006; Rip, 2009; Yesley, 2008).

One of the main differences between ELSA and ELSI is their geographical use, with ELSA being more commonly used in Europe, while ELSI is more commonly used in the US. However, in the context of healthcare, ELSI is also often used in Europe. However, both terms are often used interchangeably, and there is much overlap in their research. However, for this paper, we will use the term ELSA as our research stems from the Dutch context of ELSA labs, which is also the geographical (i.e., Europe) and contextual (i.e., AI applications) focus of this paper.

While ELSA has been around since the 1990s, there is (still) little methodological grounding of how it should be implemented – although several recent articles explore methods and ways that it can be applied in the context of AI (Juhasz et al., 2025; Ryan and Blok 2023; van Hilten et al., 2024). In practice, ELSA is often viewed as an umbrella concept that encompasses various methodologies, disciplines, and approaches for analysing ELSA aspects of current and future science and technology research. The approach typically emphasises the need to examine the societal impact of science, identify potential issues before they occur, and guide researchers on best practices according to ethical norms and legal standards. ELSA research often abides by some general guiding features (see Table 1).

**Table 1:** Four guiding features of ELSA (Ryan and Blok, 2023; taken from Zwart and Nelis, 2009)

Four Guiding Features of ELSA
1. Proximity/participation: embeddedness of ELSA research in scientific programmes
2. Anticipate: early anticipation of issues and those responsible for dealing with these issues
3. Interactivity: encourage stakeholders and the public to assume a more active role in co-designing research agendas
4. Interdisciplinarity: to bridge the boundaries between research communities such as bioethics and STS

ELSA is embedded within scientific programmes (e.g., the 4th European Research Framework Programme and the NL AI Coalition) to examine the ethical, legal, and social (or ‘societal’) aspects of scientific and technological research. The three ELSA pillars (i.e., the E, L, and S aspects) refer to what is morally right, what is recognised in law, and how it relates to society. Therefore, ELSA refers firstly to the *practice* or *behaviour* of ethical, legal, and social decision-making and conduct.

Secondly, the three ELSA pillars also refer to different aspects that are often discussed within and across these three pillars. For example, E often concentrates on a range of aspects such as (but not limited to) transparency, fairness, and autonomy, while L focuses on (but is not limited to) liability, governance, and rights, while S gives attention to (but is not limited to) broader societal aspects such as employment, power dynamics, and migration. However, ELSA research is context-specific, so no overarching ‘set’ of ELSA aspects exists. The ELSA aspects are evaluated on a case-by-case basis and are directly related to the phenomena being analysed. Because ‘aspects’ is sometimes criticised as being vague (Wang et al., 2025), ELSA aspects are sometimes defined as the ethical, legal, and social challenges and opportunities related to a phenomenon (van Hilten et al., 2024). This definition is so broad that it encompasses a wide range of values, principles, issues, and approaches. For example, van Hilten et al. (2024) outlined 23 ELSA aspects relevant to implementing AI in the agri-food sector (see Table 2).

**Table 2:** Ethical, Legal, and social aspects of AI in Agri-food

Ethical, Legal, and Social Aspects of AI in Agri-food		
Ethical	Legal	Social
Transparency	Privacy Law and Data Protection	Sustainability
Justice and Fairness	Data Ownership and Data Governance	Animal Welfare
Bias and Discrimination	Liability	Industrialisation
Beneficence	Human Rights	Impact on Gender, Class, Race
Non-maleficence	Standardisation and Protocols	Impact on Societal Views of Food Production
Freedom and Autonomy	Data and AI Regulations	Labour
Privacy	AI Code of Conduct and Guidelines	Power Asymmetries
Responsibility		Costs and Other Economics Aspects

As seen in Table 2, the aspects range from values (e.g., transparency, responsibility, and fairness) to principles (e.g., the four principles of biomedical ethics—autonomy, non-maleficence, beneficence, and justice), norms and standards (e.g., human rights, codes of conduct, and guidelines), impacts (e.g., gender, class, and race; and societal views of food production), and responses (e.g., standardisation, protocols, and regulation).

This variation corresponds to and emphasises the interdisciplinary nature of ELSA. When evaluating ethical aspects, they will often be framed and discussed differently from legal or social aspects. Ethical aspects are discussed in the context of values and principles because of the close relationship and disciplinary language used in ethical theory. The same applies to legal aspects, which are frequently addressed in legislation, regulations, and policies. Social aspects are broader because many disciplines focus on social aspects (e.g., sociology, anthropology, political science, and economics) – hence, the broader diversity of aspects outlined in van Hilten et al. (2024) under the social aspects column.

Thirdly, while ELSA refers to the adjectives ethical, legal, and social, there is also a correlation with cognate disciplines that implement ELSA research, such as ethics, law, and social sciences. However, while the pillars correspond and are often implemented by researchers in these corresponding disciplines, this is not necessarily a prerequisite – at least, it has not been emphasised as a requirement in ELSA literature (Ryan and Blok 2023; Zwart and Nelis 2009). ELSA research is interdisciplinary and can be relevant to many social science disciplines—for example, philosophy, law, sociology, political science, *and economics* (which will be the focus of this paper). Therefore, ELSA research refers to three specific foci: (1) the practices and/or behaviours found in each of the three pillars, (2) Aspects found in each of the three pillars, and (3) disciplinary examinations in each of the three ELSA pillars.

ELSA also emphasises *stakeholder engagement*. ELSA has traditionally incorporated public participation and key stakeholders to gather insights on scientific research: ‘ELSA focused on bringing the public into the discourse around how scientific and technological advancements can benefit society. It allowed the public to voice their concerns, fears, and apprehensions’ (Ryan & Blok, 2023, p. 5). This stakeholder inclusion has also been reinforced in the AI ELSA labs in the Netherlands by ensuring quadruple helix stakeholder (QH) engagement (industry, academia, civil society, and government) (NL AI Coalition 2020).

While some approaches to ELSA are starting to emphasise the importance of economics (NL AI Coalition 2020; Ryan & Blok 2023; van Hilten et al., 2024). This is mainly done in an ad hoc manner, with no precise formal placement of where and how economics should be integrated into ELSA research. It is still unclear how economics should be integrated into ELSA research. Because of ELSA’s interdisciplinary nature and the lack of clear-cut methodologies for implementation, it is sometimes unclear what the role of specific social sciences, such as economics, is in practice. Some have been explicit and proposed that it should be contained within the S pillar, as economics belongs to the domain of social sciences (van Hilten et al., 2024), while others propose that its exact placement in ELSA is still quite unclear (Ryan & Blok 2023; Zwart et al., 2014).

For this paper, we define economics in a similar way as the three ELSA pillars are defined in this section: as a behaviour or practice, as aspects, and as a discipline. Firstly, the behaviour and practice of economics are interpreted as the choices and actions of individuals and organisations within the economy, as well as the outcomes of those decisions. Secondly, economics refers to specific economic aspects or themes in a similar way to the ELSA pillars. Economic aspects can be at the micro-level (e.g., costs, efficiency, and trade-offs) or macro-level (e.g., employment, welfare, and taxation). Thirdly, economics refers explicitly to a discipline of research and the researchers (economists) within it, as well as their theoretical and empirical research on the economy.

**Fig. 1:**
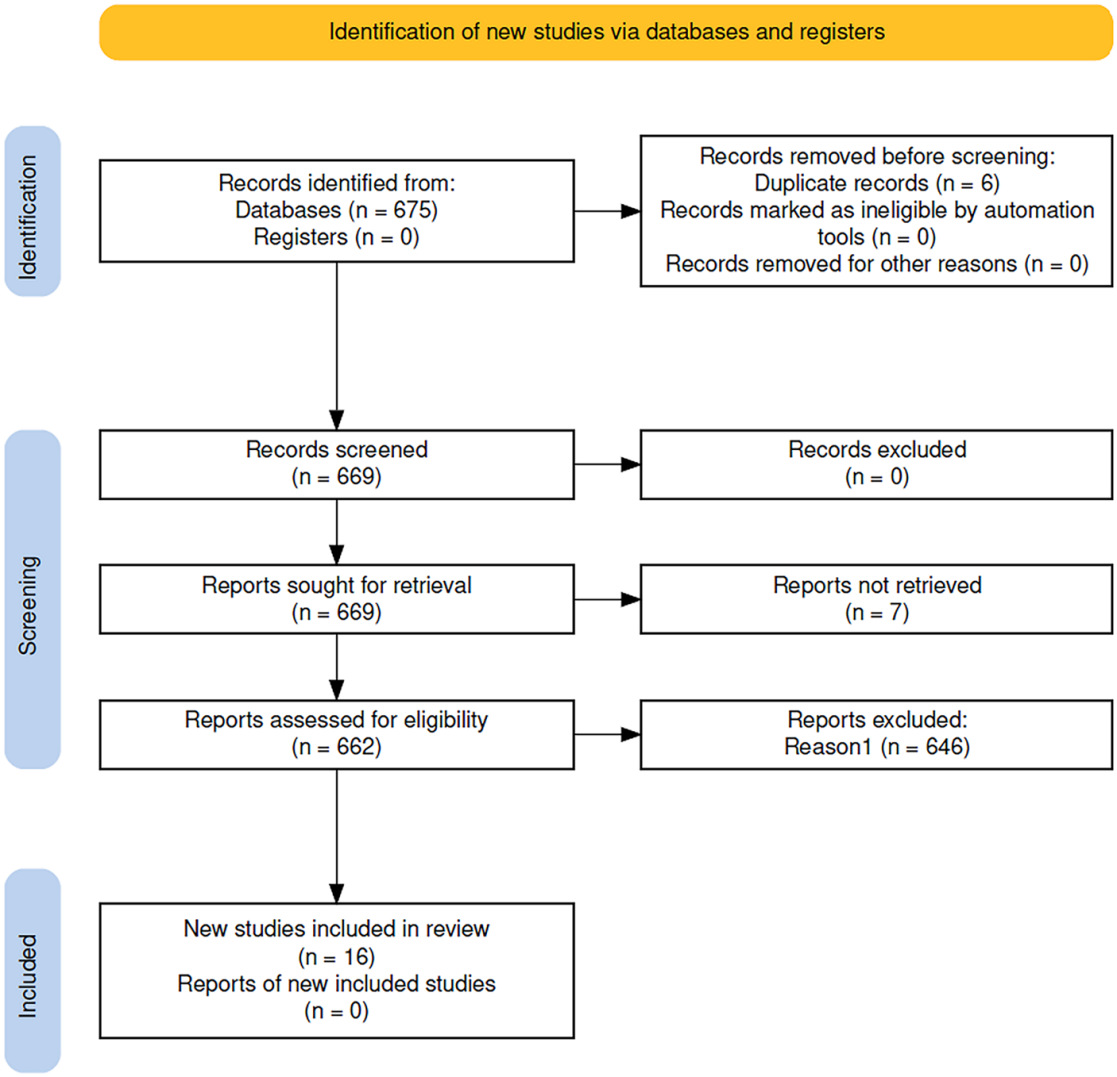
PRISMA Flow Diagram of Literature Search (PRISMA Flow Diagram used from Haddaway et al., 2022)

To identify if and how economics is currently being discussed in ELSA research, we conducted a literature search (search criteria in Appendix 1) on Scopus.^2^ This search result brought back 675 results, and duplicate articles were excluded (6), and articles that were not about the ethical, legal, and social aspects (ELSA) (646). These articles were retrieved because they referred to a person named Elsa or the abbreviations were used in other contexts (for example, many of the articles employed the English Longitudinal Study of Ageing methodology). A further seven articles were unretrievable and could not be included in the analysis. As a result, only 16 papers were suitable for analysis (see Appendix 2 for the list). See Fig. 1 below for the literature search flow.

The following three sub-sections provide an analysis of these 16 articles to identify how economics is currently being discussed in the literature. The reason for doing this is to outline different levels of inclusion (or exclusion) of economics within current ELSA research to provide a basis for how economics can be implemented in the growing area of ELSA of AI research. As will be shown below, there are three types of approaches to deal with economics in ELSA research: economics is not a part of ELSA, economics is implicit within ELSA, or economics is not a part of ELSA but should be.

### Economics is Not Included in ELSA

From the 16 papers evaluated to identify if economics or economics-related topics were discussed (e.g., budgets, affordability of research, and inclusion of industry stakeholders), ten articles did not mention anything related to economics, either implicitly or explicitly (see Åm, 2019; Christoforaki & Beyan 2024; Forsberg, 2014, 2015; Myskja et al., 2014; Pichl et al., 2023; Rip, 2009; Strech et al., 2016; Van Der Zande et al., 2011; Zwart & Nelis 2009).

A possible reason for the lack of inclusion of economics in these papers is that they were often focused on ELSA research in publicly-funded genomics projects/research (Pichl et al., 2023; Strech et al., 2016; Van Der Zande et al., 2011; Zwart & Nelis 2009), teaching or implementing ELSA in the curriculum (Christoforaki & Beyan 2024; Strech et al., 2016; Van Der Zande et al., 2011), tensions in science-society policy (Åm, 2019; Forsberg, 2014), or were quite general papers about challenges faced in ELSA research and future directions (Forsberg, 2014, 2015; Rip, 2009; Zwart & Nelis 2009).

Therefore, it may be less surprising that these articles did not focus on economics because economics may not be clearly and directly relevant to the scope of their papers. These articles did not discuss why they excluded economics or indicate whether this was intentional or unintentional. Therefore, it is difficult to infer whether these authors view economics as part of ELSA (but not the focus of their research) or whether economics is not part of ELSA (and whether it should or should not be). There is insufficient reason to conclude that economics is not a pressing issue for these researchers.

### Economics is Implicit within ELSA

Of the six remaining articles, three implicitly discuss economics (while the remaining three will be discussed in the following section). For example, the first article discusses the affordability of certain expensive biomedical technologies, making them inaccessible to many people in society (Zwart, 2012). A result of this would be that many could not afford suitable healthcare, which has implications for the freedom and autonomy of patients (Zwart, 2012).

The second article, by Alvarez et al. (2022), states that ELSA researchers should ask relevant questions in precision medicine. One out of the eight questions they pose is directly related to economic aspects: ‘Are there groups particularly affected or excluded by new technology (e.g., because of economic and educational status, gender, being part of a minority or from a disadvantaged geographical area, etc.)?’ (Alvarez et al., 2022, p. 193).

In the third article, by Jacobs et al. (2024), the authors list several questions from the three different ELSA pillars. In the social list, one of the questions focuses on economic aspects: ‘How does income, employment, housing, or education affect the construction of safety and impacts of the device? What about religious beliefs, language, business practices, social organisation, and customer preferences?’ However, this is the only mention of economics within the paper.

These three papers discuss economics scantily and mostly indirectly. However, they all refer to different economic aspects related to ELSA concerns. For example, the first paper addresses the affordability and accessibility of resources and services, and how these issues affect ELSA concerns, specifically the freedom and autonomy of *individuals*. Suppose individuals cannot afford certain goods and services. In that case, this curtails their freedom and autonomy to make decisions about their lives that they would have otherwise made, namely, accessing these goods and resources. Economic aspects have a direct impact on ELSA concerns.

The second article focuses not on individuals but on *groups*. It links economics to ELSA topics, such as discrimination and justice issues against specific groups of people. The authors ask whether or not new technologies (e.g., AI) affect or exclude groups based on their economic status. However, new technologies may also affect or exclude groups based on their educational status, being part of a minority, or residing in a disadvantaged geographical area. While economic disadvantage is not a fundamental characteristic of these groups, it has often been a considerable factor, related to other disadvantages.

The third article considers the economic aspects of *designing technology*. The authors propose including economic aspects (e.g., income, housing, employment, and education) to ensure the ‘construction of safety and impacts of the device’. While the authors are not explicit about what they mean by the safety and impacts of the device, it may relate to ELSA topics such as non-maleficence and human rights.

### Economics is Not Part of ELSA but Should be

In their paper comparing RRI and ELSA, Zwart et al. (2014) state that ELSA researchers have previously overlooked topics related to industry and economics (Zwart et al., 2014). ELSA may benefit from taking an example from RRI in its inclusion of industry: ‘RRI may increase the relevance of ELSA, as this type of research may now become part of broader transitory processes. New types of partnerships, notably with industry, will evolve, urging the introduction of new research techniques (for instance, adopted from innovation studies)’ (Zwart et al., 2014, p. 17). They state that ELSA research has often focused on the micro-level socio-economic impact of case studies. In contrast, RRI focuses on more significant macro-level socio-economic impacts and societal challenges (Zwart et al., 2014, p. 14).^3^

Ryan and Blok (2023) state that economic considerations are not inconsistent with ELSA despite often not being included. ELSA research does not purposely exclude economics from being discussed, nor does it state that there is something inherently problematic with economically benefiting from science and technology (Ryan & Blok 2023). Bos and Van Lente (2014) propose that ELSA research could benefit from incorporating more commercially embedded approaches (Bos & Van Lente 2014). Despite their rationale for being more proactive in including economics in ELSA research, Zwart et al. (2014), Bos and Lente (2014), and Ryan and Blok (2023) do not discuss how economics can be implemented in ELSA. There is a lack of clarity on how this can be implemented in practice and how economics should be applied to ELSA research.

## What Should be the Role of Economics in ELSA of AI?

The four features outlined in ELSA research (proximity, anticipation, interdisciplinarity, and interactivity) may help to provide clarity on the role of economics in ELSA of AI (Ryan & Blok 2023; Zwart & Nelis 2009).^4^ Firstly, the embeddedness of economics within ELSA research (proximity) entails ensuring that economics is closely integrated within ELSA research and projects. Secondly, there is the potential that economics is an important topic to be analysed within ELSA research, either indirectly (Alvarez et al., 2022; Jacobs et al. 2024; Zwart, 2012) or directly (van Hilten et al., 2024) (*anticipate*). Thirdly, economics can be integrated within the interdisciplinary approach proposed by ELSA by incorporating it into the research of individual researchers and their teams or by including economists within the team (*interdisciplinarity*). Fourthly, there is the possibility of including economic actors within stakeholder consultation of ELSA research (*interactivity*).

**Fig. 2:**
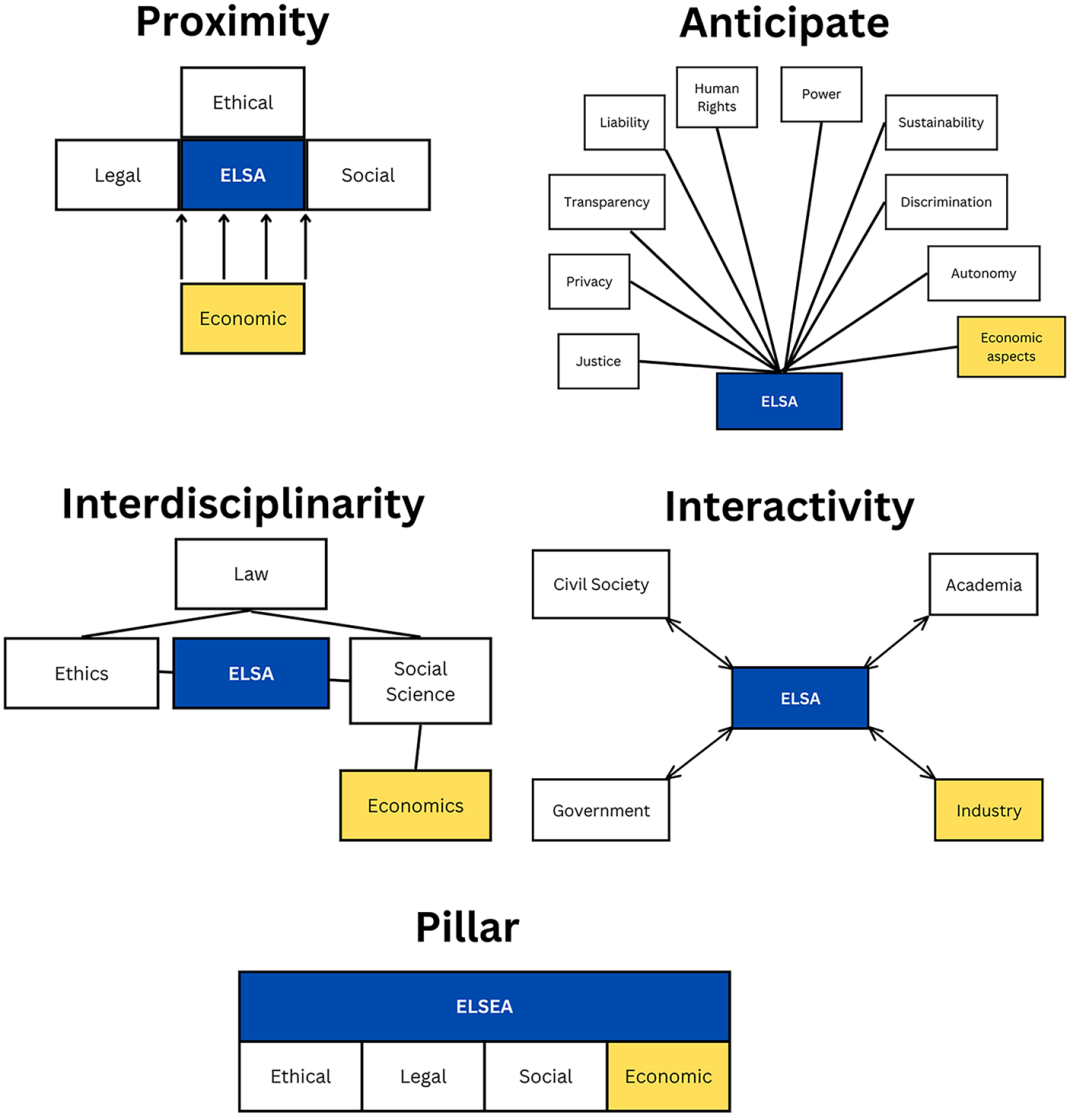
Potential Placement of Economics in the Four Features (and Pillars) of ELSA

However, in addition to these four features, there is a fifth overarching question about the placement of economics within ELSA – whether economics warrants a more formal integration in ELSA, such as creating an additional fourth pillar, making it ELSEA. The following sections examine the four possible ways to include economics in ELSA of AI, with the fifth inclusion (as a pillar) being the focus of Sect. “Economics as the Fourth Pillar of ELSA” (see Fig. 2).

### Proximity

Zwart and Nelis (2009) argued for a closer relationship between ELSA and the formal^5^ and natural^6^ sciences. Zwart and Nelis (2009) stated that closer proximity between ELSA and genomics research would allow ELSA

to stay relatively well-informed and up-to-date, but also allows the development of an anticipatory orientation that can be seen as typical of ELSA projects: studying the possible societal impact of genomics in the near or distant future, and the ways in which future agendas of genomics research can or could be shaped by broader public and societal developments. (Zwart & Nelis, 2009, p. 2)

There is a two-way interaction between ELSA researchers and genomics researchers, leading to a mutually beneficial situation where ELSA can be better informed and thus conduct more effective research. Genomics researchers can gain a deeper understanding of potential ELSA-related aspects and ways to anticipate and prevent issues from arising. Perhaps, something similar can occur between the proximity of economics and ELSA research. Closer integration of economics to ELSA could provide better insights into ELSA research(ers) and lead to improved adoption of ELSA recommendations in practice.

A common criticism of creating closer proximity between economics and ELSA is that economics is already a given within the AI industry and, thus, is unnecessary to include in ELSA of AI research. Economics is already a significant driving factor in the development of AI, so it is receiving ample attention elsewhere. The economic factors related to AI are already well-established, so there is no need for additional reflection in ELSA research.

Although it is true that economics is considered elsewhere in the AI process and already takes a prominent role, this does not necessarily mean it should be ignored in ELSA research. The fact that economics plays such an important role may be why it *should* be considered in ELSA. If one excludes economics from ELSA research, there is the possibility that ELSA results will not be taken seriously because they do not include the economic reality of the phenomena being discussed. As a result, ELSA research ‘will fall on deaf ears or remain only in the academic textbooks and journals of its niche disciplines’ (Ryan & Blok, 2023, p. 14).

However, numerous advancements have already been made in incorporating ethical, legal, and social concerns within the discipline and theory of economics over the past five + decades, with specific subfields emerging, such as the philosophy of economics, business ethics, sustainable entrepreneurship, corporate social responsibility, responsible innovation, and social innovation, to name a few. Numerous prominent journals have emerged to cater for this growing field of study, such as *Business Ethics*, *Responsible Innovation*, *Business & Society*, *Business & Society Review*, *Economics and Philosophy*, and *Social Entrepreneurship* (to name but a few). Therefore, an apparent effort is being made to bridge the gap between economic theory and disciplines commonly integrated in ELSA research (i.e., ethics and sociology), despite the lack of linkages still being made to the topic of ELSA of AI.

Additionally, if ELSA incorporates economics, it has better potential to be considered and implemented in companies’ business models, ensuring the adoption of social responsibility in the industry (Blok and Lemmens, 2015; Long and Blok, 2018).^7^ Companies require ELSA to be translated into product requirements that align with the business models to comply with ELSA while providing added value to their customers (Lubberink et al., 2017). It has been shown that incorporating social responsibility (like ELSA) may benefit companies by allowing them to anticipate risks, improve their products, ensure compliance, and improve their reputation and image (Blok et al., 2020). For example, the company Zillow used AI-driven pricing models to buy and sell homes (‘house flipping’), but their models miscalculated market fluctuations, which resulted in overpayment for properties, leading to a loss of $10 billion in market capitalisation and one in four of its employees being made redundant (CausaLens, 2021). However, if the company had implemented ELSA early on, it might have been able to identify these risks before they occurred. Emphasising values such as greater transparency and explainability of its models may have provided clarity on issues with their algorithms, allowing them to avoid the pitfalls that resulted (CausaLens, 2021).

Identifying ELSA aspects in advance may lead to better decision-making on AI and improved social and economic outcomes for the company (Long & Blok 2018). Therefore, implementing ELSA can give companies a competitive advantage while also ensuring a higher level of social responsibility. This win-win between the business case of organisations and societal considerations (referred to as ‘double materiality’ in the literature) is something ELSA may lose out on if it ignores economics (Garst et al. 2022; Ryan, Popa, et al. 2024).

Integrating ELSA into economics could lead to real-world changes in more ethical business practices and should not be overlooked. Therefore, for ELSA to be taken seriously and integrated into this process, it is best not to completely ignore the economics of the company and the industry involved. Otherwise, it will be seen as overly theoretical with no real-world implications. For example, suppose ELSA researchers only focused on the potential ELSA aspects of the AI used in the Zillow case without any additional reference to the economic implications of the company’s practice. There is a strong likelihood that focusing solely on ethical concerns would not have persuaded the company to change its practice. However, engaging with the economic repercussions would probably have strengthened the ELSA concerns about why the company should reevaluate its use of AI in this situation. Including economic aspects may support and reinforce ELSA in practice, creating a more significant societal impact than if it only focuses on non-economic concerns.

### Anticipate

Zwart and Nelis (2009) state that early anticipation of ELSA aspects is crucial for addressing potential issues in a responsible and proactive manner. Defining, anticipating, and responding to various ELSA aspects relevant to AI helps mitigate undesirable outcomes while ensuring desirable ones. ELSA research identifies ethical, legal, and social challenges and opportunities in specific contexts and applications (van Hilten et al., 2024). One may extend this argument by proposing that anticipating potential economic aspects is also essential. Economic aspects can range from micro-level concerns, such as costs, efficiency, and trade-offs, to larger macro-level concerns, including employment, welfare, and taxation. Economic aspects are crucial for avoiding pitfalls and responding to challenges responsibly, which correspond with ELSA’s ambitions and objectives.

However, a possible criticism of including economic aspects within ELSA is that economic aspects are beyond the scope of ELSA research. A proponent of this viewpoint may claim that ELSA research focuses on the ethical, legal, and social aspects of AI, whereas analysing economic aspects is too different. This difference is similar to how astronomy is beyond the scope of chemistry, or particle physics is beyond the scope of research on 16th-century conquistadors. Similarly, economics does not ‘fit’ within the scope of ELSA research.

This incommensurability can also be compared to the fact that economics lies beyond the scope of formal and natural sciences. Even if we acknowledge that economic motives nowadays incentivise science, this does not necessarily imply that economics should be part of the research. For instance, discussing economics in a paper on fundamental research on sickle-cell anaemia, nuclear fission, or the tracking of celestial objects would probably be seen as strange, unwarranted, and unscientific. Therefore, if economics is beyond the scope of scientific research, then can the same not be said about its exclusion from ELSA research? If economics is not necessarily warranted in biology, physics, and astronomy, why would it be warranted in ELSA?

Economic aspects are often closely intertwined with ELSA aspects in a much different way than formal or natural scientific research. While economics may motivate science, be essential for funding scientific research, or be necessary for accessibility to the resources/outcomes of the research, economics is much less critical (or at least should be) for the scientific validity of formal and natural science research. While these same issues also apply to ELSA research, i.e., economics may motivate ELSA research (e.g., prestige with publications or for obtaining lucrative academic positions), it is essential for funding ELSA research (e.g., appealing to specific requirements within funding calls), and may only be accessible to those who can afford it (e.g., research behind paywalls), the relationship between ELSA and economics typically goes beyond this in many different ways (see Table 3).

**Table 3:** Examples of ELSA and economic relationships

Relationship Type	Examples
Economic aspects impact ELSA	A large AI company cuts its workforce to increase profitability: - It may impact employment and community spirit (social) - It may impact the human welfare of individuals—stress and pressure to find new work (ethical). - It may also lead to increased criminality in the community because of a lack of alternative jobs (legal).
ELSA impact economic aspects	- Ethics guidelines may propose that AI companies become more transparent. Still, this could result in others using their trade secrets and using this information against them, thereby outcompeting them in the sector (ethical). - Publicly shaming AI companies with poor environmental performance may lead to widespread boycotting of their products (social). - Adherence to the GDPR costs AI companies a lot of money to change their systems and hire people to ensure compliance (legal).
ELSA underpins economic aspects	- The increased price of using AI technology has led to some being unable to afford it and, thus, using it (e.g., a justice issue) (ethical). - There is an increase in emissions caused by the profit from developing AI (e.g., a sustainability issue) (social). - AI companies benefit from their recommendation systems but claim that users are responsible for their own decisions (e.g., a liability issue) (legal).
Economic aspects underpin ELSA	- A social media platform opts to use algorithms that create more engagement (and profit) despite the social tensions they create (e.g., cost-benefit analysis) (social). - A government implements AI facial recognition in public places to reduce crime, which some claim infringes on freedom. The government states it increases the welfare of the population (e.g., welfare economics) (ethics). - AI companies sell customers’ data because it is profitable despite contravening regulations. Even if they get caught, the benefits outweigh the potential fines (e.g., the game theory approach) (legal).

This table illustrates that ELSA and economic aspects are often profoundly connected, and discussing one often precludes discussing the other. However, this does not mean that all economic aspects are relevant for ELSA or that all ELSA is relevant for economics. This also relates to the fact that not all economists will find ELSA relevant for their research, and not all ELSA researchers will find economics essential for theirs, too. Instead, the table highlights how ELSA aspects and economic aspects are sometimes inextricably linked to find where there is overlap and the need to engage with other disciplines, researchers, and aspects outside of one’s field.

This overlap can be seen in a recent paper that comprehensively maps ELSA aspects of AI in the agri-food domain (van Hilten et al., 2024). The authors aimed to identify the most relevant ELSA aspects for the agri-food domain. However, because economic aspects were so profoundly tied to the social aspects within the literature, they proposed creating an explicitly economic aspect (‘costs and other economic aspects’) and several socio-economic related aspects (e.g., industrialisation, labour, and power asymmetries) (see Table 2 earlier).

This section highlighted the importance of including economic aspects within ELSA research analysis. However, it remains unclear whether this requires the inclusion of economists within the interdisciplinary approach commonly proposed in ELSA research. The following section will examine how the discipline of economics should be understood within ELSA research.

### Interdisciplinarity

ELSA brings together different disciplinary experts to ‘join forces’ to respond to the societal impacts of technology (Zwart & Nelis 2009). While individual disciplines have different strengths in analysing AI’s societal impacts, interdisciplinary teams have great potential to address these challenges more holistically. Due to its interdisciplinary nature, ELSA is often viewed as an umbrella concept that encompasses various methodologies and approaches implemented by researchers from diverse disciplinary backgrounds (Wang et al., 2025). This point is evident in the 25 funded AI ELSA labs in the Netherlands, which comprise a diverse range of researchers from various backgrounds in art, media, psychology, computer science, anthropology, ethics, law, and sociology (Wang et al., 2025). ELSA’s approach toward analysing phenomena is interdisciplinary in theory (Ryan & Blok, 2023; Zwart et al., 2014) and deployment (NL AI Coalition, 2020; Wang et al., 2025).

This interdisciplinarity is often evident in the context of the three pillars of ELSA. For example, while ethical aspects could be linked to the discipline of philosophy, with its sub-discipline of ethics, analysing ethical aspects also takes place in other fields. For example, the emerging field of ‘AI ethics’ contains researchers from psychology, political science, philosophy (and economics) (Cocchiaro et al., 2024; Hagendorff, 2023; van Hilten et al., 2024). Similarly, focusing on legal aspects is not *confined only* to law faculties. The study of legal aspects also often plays a substantial role in political science and sociology (and economics). Lastly, social aspects are so broad that they do not have a specific disciplinary tie and are often analysed in disciplines as diverse as sociology, psychology, anthropology, political science, and economics. Therefore, the analysis of ELSA aspects is often undertaken within and by many social science disciplines and researchers.^8^

While trained scholars in various fields often conduct ELSA, they have historically come from philosophy (e.g., ethics), law (e.g., law), and social sciences (e.g., sociology). Therefore, one may argue that expecting these kinds of ELSA researchers to become knowledgeable about economics may be unfair because it is outside their scope of expertise and research fields. However, ELSA research typically implies an interdisciplinary approach, which can be implemented in several ways and could afford the inclusion of economics. This inclusion could come in the following ways: ELSA researchers conduct interdisciplinary research (thus including the analysis of economic aspects), continue to specialise in their respective fields while being part of interdisciplinary teams (including economists into the team to analyse economic aspects), or become more interdisciplinary while also being part of an interdisciplinary team (where researchers and their teams examine economics).

Firstly, if researchers integrate interdisciplinary approaches, they could also include an examination of economic aspects (e.g., as seen in Table 3). However, this is not to say that an ethicist must become an economist, just as they would not be expected to become a lawyer. To make a comparison, for an ethicist to conduct ELSA research on privacy ethics in AI, it would be good practice to be familiar with privacy law. This does not imply that they need specific legal training or must become GDPR experts. However, conducting good interdisciplinary ELSA research on privacy would require engaging with discourse on the same topic in neighbouring social science disciplines, or at least not actively ignoring it.

The same can be said of including economics within ELSA research. It is not required to become an economist to engage with economic aspects. However, one should acknowledge and understand economic aspects relevant to the topic or social phenomena being analysed. For example, if someone is examining justice-related aspects in the context of AI, then it would seem relevant to also include potential injustices caused by economic aspects, such as the affordability and accessibility of that technology or how its use may create poverty for some, or that it is biased against people of lower economic status (see examples in Sect. “Economics is Implicit within ELSA”).

If researchers want to focus on discipline-specific research, another option is to ensure that interdisciplinarity is part of the ELSA team, which is already being done in practice. There are large teams of researchers exploring different domains in the 25 Dutch AI ELSA labs. These interdisciplinary teams comprise experts from diverse fields, including computer science, psychology, philosophy, law, and sociology. Many of these teams consist of up to 20 researchers (Brightlands Smart Services Campus, 2024), so including an additional economist or someone engaged with the economic aspects of AI in the project may not be a huge request to implement. Having such a high capacity of researchers working on such large ELSA projects while ignoring a topic as important as economics may be an oversight.

Likewise, it may be interesting for economists to participate in ELSA of AI labs as part of their research. There are already many economists working at the intersection of philosophy, ethics, and sociology *of economics* (and also the economics *of AI*), so it is not beyond the scope of imagination that they would be internally motivated to participate and become involved in such projects and labs. The type of economic expertise needed in each ELSA lab would depend on the types and context of the AI applications being assessed. Therefore, there is no single blueprint of an economist required (for example, they could be behavioural, environmental, development, or financial economists, to give a few examples) in the same way that different types of philosophers, legal scholars, or sociologists would be required, depending on the scope of the project or lab.

Overall, economics can be integrated into the interdisciplinary nature of ELSA research in many ways. Their inclusion in interdisciplinary ELSA of AI research may, in fact, have a formative effect on these researchers through their collaboration with other social scientists on these topics. It may lead to greater consideration and inclusion of ethical, legal, and social concerns in the field of economics as a result. While ethical, legal, and social scholars may also benefit from this interdisciplinary collaboration by gaining greater insights into patterns, modes of thought, and economic drivers behind AI development.

### Interactivity

Interactivity focuses on including different groups and stakeholders within the ELSA research process. These stakeholders include citizens, policymakers, civil society organisations, AI developers, AI companies, and researchers (Ryan, De Roo, et al. 2024). It is essential to include different stakeholders to retrieve input and feedback on AI that will, directly and indirectly, impact their lives. It is also essential to identify potential opportunities and challenges of AI in individuals’ lives. ELSA research proposes to go beyond simply desk research and engage with the communities that technologies (such as AI) will affect: ‘Societal interaction is not something that can be set apart from societal research in a strict sense: it must be an integrated part of the research from the outset’ (Zwart & Nelis, 2009, p. 3).

ELSA researchers should interact with different stakeholders throughout their projects as part of this integration. This interaction can occur through focus groups, scenario workshops, interviews, public outreach, and collaborative dialogues to understand how stakeholders perceive ELSA aspects of AI (Wang et al., 2025). Stakeholders are expected to benefit from this interaction by broadening and deepening their knowledge of the topic and being more informed about ELSA (Zwart & Nelis 2009). Participation aims to create a dialogue to help shape scientific research and ‘help shape policy-making through the early identification of social, legal and ethical issues’ (Zwart & Nelis, 2009, p. 3).

However, what role does economics have in this interactivity? Should specific economically-focused stakeholders be included in this participation, and what should this look like in practice? In the original account of interactivity, Zwart and Nelis (2009) refer to citizens, policymakers, non-governmental organisations (NGOs), and medical professionals. However, there is no clear indication of industry stakeholders. One possible reason is that ELSA initially focused on ‘public’ research (e.g., genomics and research at universities) (Rip, 2009; Zwart & Nelis 2009).

If we compare this to current trends, most AI research currently takes place in the private sector, which has much more room for investment than public funding schemes and universities. Therefore, the importance of including industry stakeholders in ELSA research must be acknowledged. While many earlier formulations of ELSA did not include the participation of industry stakeholders, there appears to be a shift in focus in ELSA AI labs.

Industry stakeholders are often included in the interactivity process of ELSA through the quadruple helix (QH) approach. QH refers to stakeholders from industry, government, civil society, and academia (Carayannis and Campbell 2010; 2009; Miller et al., 2018). ELSA research can benefit from including ‘industrial partners, and economic actors have the incentive to translate responsibility into marketable products that have added value for consumers’ (Ryan & Blok, 2023, p. 14). This inclusion allows for better reflection on economic aspects with industry stakeholders and how ELSA can be incorporated within private-funded AI research and development (R&D) (Ryan and Blok 2023). However, even when ELSA projects focus on the public deployment of AI, there may still be a strong reason not to overlook the economic impacts of their research. Zwart et al. (2014) emphasise the importance of including economics within ELSA because

society should have a say in how the money is invested, i.e. trans-mutated into knowledge (which, hopefully, and eventually, can be trans-mutated back into money once again, through a process which is now often referred to as ‘valorisation’). Moreover, society will also function as the future consumer of the scientific knowledge thus produced, and as the future market for new technological devices. (Zwart et al., 2014, p. 9)

In addition to industry stakeholders discussing economic aspects, it is expected that economics will also be a significant topic of conversation among governmental actors, but from a macroeconomic perspective rather than the microeconomic focus of the industry stakeholders. This was also exemplified in a recent study that analysed the ethical, legal, social, and environmental aspects of AI in four workshops with 32 AI professionals from the four QH groups (Ryan, et al. 2024). While the workshop hosts did not mention economics in their preliminary presentation, nor was it a focus of the conference (or workshops), it was the most discussed topic throughout the workshops with the participants (from government, civil society, industry, and academia) emphasising the importance of including micro and macro-level economic aspects. This study concluded that it is naïve and undesirable to focus solely on ELSA aspects of AI while excluding economic considerations, and that the importance lies in including diverse QH stakeholders to uncover economic aspects related to AI.

In addition, ELSA research could give more attention to the structures, incentives and goals of the different QH stakeholder groups and obtain input from the economic sciences to help understand these developments and provide ways to overcome hurdles for development and implementation. This reiterates the importance of including economists in large ELSA lab teams, as mentioned in Sect. “Interdisciplinarity”.

## Economics as the Fourth Pillar of ELSA

After reviewing the four features and how economics should be integrated within ELSA of AI, we are still left with the elephant in the room—should economics be included as a fourth pillar of ELSA? If economics can and should be integrated within ELSA through the ways outlined in the previous four subsections, does this not warrant its inclusion as a fourth pillar? Economics is already the leading driver for AI development, deployment, and use in current research on AI. In addition, the previous sections have demonstrated the importance and necessity of incorporating economic aspects, economic stakeholders, and economic disciplinary knowledge into ELSA research to ensure its effective implementation. Therefore, because economics already plays a prominent role in AI, as shown in the previous sections, it should be given greater attention in ELSA research. Perhaps it needs to be formally recognised as a fourth pillar.

While some scholars have already included economics as a fourth pillar in ELSA (Benefo et al., 2022; Spindler et al., 2020), it is often added without much explanation or justification. Therefore, this section addresses three criticisms of including economics as a fourth pillar to evaluate whether its inclusion is warranted.

### Including Economics will Water Down the Other Pillars and ELSA as a Whole

The first argument is that including economics as a fourth pillar in ELSA research would weaken the other pillars and the overall approach of ELSA. If we include economics as the fourth pillar in ELSA, there is the potential that its inclusion would water down the impact and message of ELSA research (Ryan & Blok, 2023, p. 13). Suppose economics is included as a fourth pillar. In that case, the ethical, legal, and social aspects are given reduced importance because each pillar is reduced to one-quarter, as opposed to one-third, of an approach.

However, it is unclear why including economics as a fourth pillar would water down ELSA research in practice. As demonstrated earlier in the identification and response to ELSA aspects (anticipation), many ELSA aspects are closely tied to economics and vice versa. It was also shown that economics as a social science discipline is closely related to those focused on ELSA research (proximity). Both approaches examine social phenomena, human behaviour, and societal impacts within their research. It was also demonstrated that it is often essential to involve economic stakeholders to gain a more comprehensive understanding of AI and how ELSA can be effectively integrated into an industry setting.

Therefore, there are many strong reasons for including economics within ELSA research, and much effort has already been made for implementing it in practice (e.g., implicitly within scholarly research (see Sect. “Economics is Implicit within ELSA”) or in projects (see the ELSA lab examples in Sect. “Interactivity”)). However, if researchers are tasked with implementing a fourth pillar in ELSA, they must formally include economics in ELSA research. This inclusion may be an additional burden for individual researchers. Still, as mentioned earlier, this is no different from an ethicist having to become familiar with the law or a lawyer being required to understand social theory. As ELSA focuses on the social phenomena of AI through interdisciplinary investigation, it is unclear why including economics as a fourth pillar would water down or harm ELSA research or overburden ELSA researchers. Therefore, this argument is not credible for excluding economics as a fourth pillar in ELSA.

### Economics will Dominate the Debate if Included

A second possible criticism is that including economics in ELSA research could dominate the debate. While there are many different frameworks and theories within economics, some strands of it are seen as amoral or immoral (Prasch, 2003). For example, Milton Friedman was renowned for stating that the primary social responsibility of businesses is to increase profits (Friedman, 1970). Many subfields within economics incorporate ethical, legal, and social concerns (e.g., business ethics, corporate social responsibility, and socially responsible entrepreneurship), but if economics is included as a fourth pillar, it may dominate the ELSA debate. Economic aspects will be pushed to the forefront and given greater priority, while the importance of the other aspects (namely, the original ELS aspects) will be negated.

However, it is unclear why the greater formalisation of its role in ELSA (i.e., integration as a fourth pillar) would somehow make it dominate ELSA debates. Despite this, giving more significant emphasis on economic concerns may still risk allowing unethical approaches toward AI to continue and even lead to a type of ‘ELSA-washing’ if implemented inappropriately, where companies can use it to promote that they are ‘ELSA-proof’ while continuing business as usual. This issue must be addressed in any socio-ethical approach by developing safeguards and potential ‘red lines’ within the approach and ensuring that guidelines and recommendations are adopted from the ELSA research. However, it is not necessarily a fundamental critique against including economics as a fourth pillar in ELSA.

### It Would Create A Slippery Slope for Additional Pillars

Thirdly, including economics as a fourth pillar might create a slippery slope that requires other social sciences from the S pillar to be included as additional pillars. If economics is included, why not include historical, geographical, or political pillars? Many of these areas are already being explored in the analysis of ELSA of AI, similar to how we proposed addressing economic aspects in ELSA (as described in Sect. “Anticipate”). For instance, when discussing an impoverished or disadvantaged group, one may need to consider other research areas to understand better or explain those ELSA aspects. Factors such as the location of that group (geographical), impacts of colonialism (historical), or religious persecution (theological and/or political) may be factors to consider when discussing ELSA aspects such as justice and discrimination. Simply focusing on ELSA aspects (e.g., justice) without engaging in these other areas would provide an incomplete analysis of the topic.

Suppose economics is included as a fourth pillar in ELSA, and these other areas are related to ELSA research similarly to economics. In that case, it begs the question: Why not include these as additional pillars as well? Therefore, including economics may create a slippery slope where researchers constantly add pillars to ELSA, altering its name and approach and diverting it from its original core meaning. This addition may confuse and alter the main message and approach, which is understood as ‘ELSA’.

It may be worthwhile to reflect on another community whose group (and abbreviation) has expanded over the past several decades. During the 1990s, lesbian, gay, and bisexual activists created a community, adopting the initials LGB to create greater solidarity and recognition. Later, trans and queer were added as the fourth and fifth pillars of the community, respectively. Following this, several variations and alterations (e.g., LGBTQ+, LGBTQIA, and LGBTQIA+) have been introduced to include a greater diversity of genders and groups. These alterations are because the added groups did not feel adequately represented in the earlier formulations of the group. However, the focus of the community did not change as its core message is still to create unity and representation of traditionally marginalised groups because of their gender, sex, or sexual preferences.

The inclusion in the community has been fluid. It will probably continue to adapt and change in response to those communities that are already well-represented in society (e.g., heterosexual, hetero-romantic, cisgender, or endosex). Therefore, these well-represented groups would never be included in LGBTQ because the approach would lose all meaning. It was intended to be a representative community for those not traditionally well-represented, so it would not make sense to include groups that are already well-represented within society. If these well-represented groups were included, then the LGBTQ community would lose all meaning and relevance. It would no longer be a representative community of marginalised voices but would become so broad as to include everyone within society.

This argument aligns with the inclusion of economics as a fourth pillar of ELSA. There may be adequate justification for including additional pillars in ELSA research if those pillars are inadequately represented within mainstream science and R&D and fit within the objectives of ELSA research. Therefore, there is not necessarily a reason for not including additional pillars if they still fit within the overall scope and objective of ELSA research. However, where things become more problematic for the inclusion of *economics* as a fourth pillar in ELSA is that it would make ELSA, in the same way that including heterosexuals or cisgenders would make the LGBTQ community, meaningless.

ELSA was initially created because ethical, legal, and social aspects were *not* included within science and R&D, while economics has typically been the most significant topic leading the debate. ELSA research was created to respond to and give greater attention to the needs of ELSA aspects within research. Traditionally, there has been an overemphasis on scientific, technological, and economic concerns in the area, while ELSA has been overlooked or ignored. Therefore, adding economics as a fourth pillar would contravene the fundamental raison d’être for ELSA’s existence and go against its core objectives to give attention to aspects that have traditionally been overlooked.

Therefore, economics is essential for understanding and engaging with the development and implementation of AI systems in practice. While considering economic aspects and their relationship to ELSA aspects, including industry stakeholders, and having ELSA researchers engage with economics topics is essential, economics as a fourth pillar in ELSA is neither necessary nor desirable.

## Conclusion

This paper focused on the question: What is the role of economics in the ELSA of AI research? We used the four features of ELSA (Zwart & Nelis 2009) to demonstrate how economics can, and often should, be included within the proximity, anticipation, interdisciplinarity, and interactivity of ELSA research. When analysing whether economics should be a fourth pillar of ELSA, it became clear that many common arguments against its inclusion (e.g., that it would water down the other pillars or dominate the debate) were insufficient. However, it was shown that economics should not be included as a fourth pillar in ELSA because it would distort its meaning so much as to make the approach meaningless and contradictory to its raison d’être.

This paper demonstrates how and why economics should be taken more seriously within ELSA, as well as the ethics of AI research. It highlights many ways to retain ELSA’s core message and objectives. Future ELSA and ethics of AI research can build upon the findings in this paper and implement these approaches in practice. The active inclusion of economic aspects within ELSA and the ethics of AI research allows researchers to engage with practical challenges faced when translating and integrating ELSA in AI industry settings. ELSA and the ethics of AI researchers should not completely disregard economic realities, as it does a disservice to the potential to bring about significant change.

This paper highlighted the broad parameters of how economics can and should be incorporated into ELSA research; however, further research is needed on how exactly this can and should be implemented in practice. Specifically, economic aspects should be evaluated when conducting ELSA research; however, ELSA researchers need to know what economic aspects to look for, how to identify them in their research, and how to evaluate and prioritise them alongside other ELSA aspects. This requires further examination. However, this arguably applies to ELSA research as a whole because, as pointed out in recent literature (Wang et al., 2025), there is still a lack of clear methodological implementation and investigation of ELSA aspects.

While this paper was also focused on identifying how economics is currently incorporated in ELSA research and proposing why and how it should be included in ELSA of AI research, more research needs to take place on what exactly is expected from economists when conducting ELSA research and participating in ELSA research teams. Different economic schools of thought will also bring their own differences and idiosyncrasies to these emerging ELSA lab teams; for example, a neoclassical, Marxian, or Keynesian economist will all have very different perspectives and approaches to ELSA research. In addition, further research is needed to identify the types of economic research that can help conduct ELSA of AI research. This could range from, but is not limited to, analysing the impact of market forces/behaviour of different AI technologies on society, conducting behavioural economics analyses of individual and group behaviour, or conducting health technology assessments.

Overall, this paper has demonstrated that economics should be considered and incorporated within the ELSA of AI research, as well as the ethics of AI research more broadly, to create significant societal change and impact in the years to come. It has provided a theoretical argument for how economics can be incorporated within ELSA of AI research, but further work needs to be done to identify how this can be realised in practice. Steps need to be taken to initiate it into the ELSA of AI, such as the 30 ELSA of AI labs in the Netherlands.

## Appendices

### Appendix 1: Literature Review Search Query

(TITLE-ABS-KEY (“elsa”) AND TITLE-ABS-KEY (ethic*) OR TITLE-ABS-KEY (moral) OR TITLE-ABS-KEY (social) OR TITLE-ABS-KEY (society) OR TITLE-ABS-KEY (societal) OR TITLE-ABS-KEY (legal) OR TITLE-ABS-KEY (law) OR TITLE-ABS-KEY (economic)) AND PUBYEAR > 1993 AND PUBYEAR < 2025 AND (LIMIT-TO (LANGUAGE, “English”)) AND (LIMIT-TO (DOCTYPE, “ar”) OR LIMIT-TO (DOCTYPE, “cp”) OR LIMIT-TO (DOCTYPE, “ch”) OR LIMIT-TO (DOCTYPE, “re”) OR LIMIT-TO (DOCTYPE, “ed”) OR LIMIT-TO (DOCTYPE, “bk”)).

### Appendix 2: 16 Articles Used in Literature Review

**Table:** 

Alvarez, M. J. *R*., Griessler, E., & Starkbaum, J. (2022). Ethical, Legal and Social Aspects of Precision Medicine. In Precision Medicine in Clinical Practice (pp. 179–196). Springer.
Åm (2019). Limits of decentered governance in science-society policies. Journal of Responsible Innovation, 6(2), 163–178. 10.1080/23299460.2019.1605483
Bos, C., & Van Lente, H. (2014). Value Chain Responsibility in Emerging Technologies. In S. Arnaldi, A. Ferrari, P. Magaudda, & F. Marin (Eds.), Responsibility in Nanotechnology Development (Vol. 13, pp. 129–141). Springer Netherlands. 10.1007/978-94-017-9103-8_8
Christoforaki, M., & Beyan, O. D. (2024). Towards an ELSA Curriculum for Data Scientists. AI, 5(2), 504–515. 10.3390/ai5020025
Forsberg, E.-M. (2014). Institutionalising ELSA in the moment of breakdown? Life Sciences, Society and Policy, 10(1), 1–16.
Forsberg, E.-M. (2015). ELSA and RRI – Editorial. Life Sciences, Society and Policy, 11(1), 2. 10.1186/s40504-014-0021-8
Jacobs, G., Houdt, F. V., & Coons, G. (2024). Studying Surveillance AI-cologies in Public Safety: How AI Is in the World and the World in AI. Surveillance & Society, 22(2), 160–178. 10.24908/ss.v22i2.16104
Myskja, B. K., Nydal, R., & Myhr, A. I. (2014). We have never been ELSI researchers – there is no need for a post-ELSI shift. Life Sciences, Society and Policy, 10(1), 9. 10.1186/s40504-014-0009-4
Pichl, A., Ranisch, R., Altinok, O. A., Antonakaki, M., Barnhart, A. J., Bassil, K., Boyd, J. L., Chinaia, A. A., Diner, S., Gaillard, M., Greely, H. T., Jowitt, J., Kreitmair, K., Lawrence, D., Lee, T. N., McKeown, A., Sachdev, V., Schicktanz, S., Sugarman, J., … Árnason, G. (2023). Ethical, legal and social aspects of human cerebral organoids and their governance in Germany, the United Kingdom and the United States. Frontiers in Cell and Developmental Biology, 11. 10.3389/fcell.2023.1194706
Rip (2009). Futures of ELSA. EMBO Reports, 10(7), 666–670.
Ryan, M., & Blok, V. (2023). Stop re-inventing the wheel: Or how ELSA and RRI can align. Journal of Responsible Innovation, 1–19.
Strech, D., Hirschberg, I., Meyer, A., Baum, A., Hainz, T., Neitzke, G., Seidel, G., & Dierks, M.-L. (2016). Ethics Literacy and “Ethics University”: Two intertwined models for public involvement and empowerment in bioethics. Frontiers in Public Health, 3, 287.
Van Der Zande, P., Waarlo, A. J., Brekelmans, M., Akkerman, S. F., & Vermunt, J. D. (2011). A Knowledge Base for Teaching Biology Situated in the Context of Genetic Testing. International Journal of Science Education, 33(15), 2037–2067. 10.1080/09500693.2010.525797
Zwart (2012). Ethical expertise in policy. In Encyclopedia of Applied Ethics: Volume 1–4, Second Edition. Oxford: Elsevier. https://repository.ubn.ru.nl/handle/2066/93841
Zwart, H., Landeweerd, L., & Van Rooij, A. (2014). Adapt or perish? Assessing the recent shift in the European research funding arena from ‘ELSA’to ‘RRI’. Life Sciences, Society and Policy, 10(1), 1–19.
Zwart, H., & Nelis, A. (2009). What Is ELSA Genomics? Science and Society Series on Convergence Research. EMBO Reports, 10(6), 540–544.

## Data Availability

N/A.

## Footnotes

1 This was calculated based on an average of 20 researchers affiliated with each lab, currently, with a total of 30 ELSA labs being underway by 2027.

2 This search was conducted on September 1st, 2025.

3 However, some claim that RRI literature did not pay sufficient attention to these micro-level socio-economic impacts (Blok & Lemmens 2015).

4 However, the proximity feature has a less direct translation for the topic at hand because proximity refers to the closeness and integration of ELSA research into formal and natural science projects it is analysing.

5 For example, mathematics, logic, computer science, and statistics.

6 For example, biology, physics, astronomy, and chemistry.

7 This point has already been extensively discussed in the literature in many different approaches that emphasise the importance of including ELSA-like considerations into the industry, such as ‘responsible research and innovation’ (Martinuzzi et al., 2018; Van de Poel et al., 2017), ‘corporate social responsibility’ (Schwartz 2011; Sims, 2003; Trong Tuan, 2012), or ‘social entrepreneurship’ (Praszkier & Nowak 2011). This can be done at any life stage of the business, as well, such as in the start-up phase (Long et al., 2020; Ryan, Popa, et al. 2024; Ryan et al., 2023; Veloudis et al., 2025).

8 This point can also be said for economics. While economics is a fundamental theoretical discipline, many sub-fields within economics integrate different disciplines and approaches. For example, behavioural economics is heavily influenced by psychology and anthropology; rational choice theory is underpinned by philosophical theory and political science; and welfare economics and cost-benefit analysis is often seen as the culmination of the ethical framework ‘utilitarianism’. This close relationship is partly because economics is a specific discipline *within* the social sciences—which ELSA focuses on broadly —and may offer many insights into responding to specific ELSA aspects in practice.
